# Effects of Chk1 inhibition on the temporal duration of radiation-induced G2 arrest in HeLa cells

**DOI:** 10.1093/jrr/rru038

**Published:** 2014-06-03

**Authors:** Kamrun Nahar, Tatsuaki Goto, Atsushi Kaida, Shifumi Deguchi, Masahiko Miura

**Affiliations:** Section of Oral Radiation Oncology, Department of Oral Health Sciences, Graduate School of Medical and Dental Sciences, Tokyo Medical and Dental University, 1–5–45 Yushima, Bunkyo-ku, Tokyo 113–8549, Japan

**Keywords:** Chk1, Chk1 inhibitor, cell cycle, radiation, G2 arrest, fluorescent ubiquitination-based cell-cycle indicator (Fucci)

## Abstract

Chk1 inhibitor acts as a potent radiosensitizer in p53-deficient tumor cells by abrogating the G2/M checkpoint. However, the effects of Chk1 inhibitor on the duration of G2 arrest have not been precisely analyzed. To address this issue, we utilized a cell-cycle visualization system, fluorescent ubiquitination-based cell-cycle indicator (Fucci), to analyze the change in the first green phase duration (FGPD) after irradiation. In the Fucci system, G1 and S/G2/M cells emit red and green fluorescence, respectively; therefore, G2 arrest is reflected by an elongated FGPD. The system also allowed us to differentially analyze cells that received irradiation in the red or green phase. Cells irradiated in the green phase exhibited a significantly elongated FGPD relative to cells irradiated in the red phase. In cells irradiated in either phase, Chk1 inhibitor reduced FGPD almost to control levels. The results of this study provide the first clear information regarding the effects of Chk1 inhibition on radiation-induced G2 arrest, with special focus on the time dimension.

## INTRODUCTION

Enhancement of radiation-induced cell killing by Chk1 inhibition has been observed in several studies [1]. In most tumor cells with deficient p53 pathways, DNA damage is repaired mostly during G2 arrest [2]; therefore, releasing this arrest by inhibiting the Chk1 pathway is predicted to enhance cell killing. To date, however, little clear information has been available regarding the effects of Chk1 inhibition on the time dimension of G2 arrest, because most previous studies were based on flow-cytometric analysis, which makes it impossible to track cell-cycle changes in individual living cells.

Sakaue-Sawano *et al*. developed a cell-cycle visualization system called fluorescent ubiquitination-based cell-cycle indicator (Fucci), which takes advantage of the cell cycle-dependent ubiquitination of Cdt1 and Geminin [3]. In this system, G1 and S/G2/M cells emit red and green fluorescence, respectively; more precisely, early G1-phase cells emit no fluorescence, and early S-phase cells emit both fluorescence types [3]. In previous studies, we reported that radiation-induced G2 arrest kinetics can be visualized via the accumulation of green cells and the re-appearance of red fluorescent cells in HeLa cells expressing Fucci [4, 5]. We predicted that application of this system would allow us to obtain precise information regarding the duration of the G2 arrest. Furthermore, this system has made it possible to perform differential analysis of cells receiving irradiation in the red or green phase. In this paper, we demonstrate for the first time that Chk1 inhibitor remarkably reduces the duration of radiation-induced G2 arrest, regardless of cell-cycle phase at the time of irradiation.

## MATERIALS AND METHODS

### Cell lines and culture conditions

HeLa cells expressing the Fucci probes (HeLa-Fucci) were provided by the RIKEN BRC through the National Bio-Resource Project of MEXT, Japan. Cells were maintained in DMEM (Sigma-Aldrich, St Louis, USA) supplemented with 10% fetal bovine serum and penicillin–streptomycin, at 37°C in a 5% CO_2_ humidified atmosphere.

### Drug treatment and irradiation

Cells were treated or untreated with 300 nM Chk1 inhibitor (PF-00477736) (Sigma-Aldrich) or 60 nM Wee1 inhibitor (MK-1775) (Axon Medchem, Groningen, Netherlands) for 10 min, and then irradiated at a dose of 10 Gy in an RX-650 Cabinet X-Radiator system (130 kVp, 5 mA, 0.5 mm Al filtration, 0.895 Gy/min) or a CellRad X-irradiation system (150 kVp, 5 mA, 0.5 mm Al filtration, 2.074 Gy/min) (Faxitron, Lincolnshire, IL, USA). At the indicated times after treatment, cells were subjected to fluorescence microscopy or prepared for Western blotting.

### siRNA transfection

Twenty-four hours before transfection, HeLa-Fucci cells were seeded in growth medium. Chk1 siRNAi (Chk1 Silencer^®^ Select Validated siRNA) or Scramble siRNA (Silencer^®^ Select Negative Control No. 1 siRNA) (Life Technologies, Carlsbad, USA) duplex was mixed with Opti-Mem^®^ I Medium (Life Technologies) and Lipofectamine^®^RNAiMAX Reagent (Life Technologies); the final concentration of nucleic acid in the transfection mix was 5 nM. After incubation for 24 or 48 h, cells were subjected to fluorescence microscopy or prepared for Western blotting at the indicated times.

### Fluorescence imaging

Fluorescence images were taken using a BIOREVO BZ-9000 fluorescence microscope (KEYENCE, Osaka, Japan). For time-lapse imaging, cells were held in an incubation chamber at 37°C in a humidified atmosphere containing 5% CO_2_ (Tokai Hit, Fujinomiya, Japan). Pedigree analysis was performed according to the time-lapse imaging data.

### Western blotting

Chk1, Chk1 phosphorylated at Ser345 (p-Chk1^S345^) or Ser296 (p-Chk1^S296^), Cdc2 phosphorylated at Tyr15 (p-Cdc2^Y15^), and β-actin were detected by Western blotting. Briefly, cells were lysed using the Mammalian Protein Extraction Reagent (M-PER) (Pierce, Rockford, USA), and equal amounts of protein from cell lysates were separated using SDS-PAGE. Proteins were transferred to nitrocellulose membranes, and the membranes were blocked in 2% ECL advance blocking agent (GE Healthcare, Uppsala, Sweden) in Tris-buffered saline with Triton X-100. Proteins were detected using specific primary antibodies against Chk1 (Sigma-Aldrich), p-Chk1^S345^, p-Chk1^S296^, p-Cdc2^Y15^ (Cell Signaling Technology, Danvers, MA, USA), and β-actin (clone C4; Millipore, Billerica, MA, USA). Specific proteins were visualized using secondary antibodies conjugated with horseradish peroxidase (Santa Cruz Biotechnology, Dallas, USA) and the ECL Western Blotting Detection reagents (GE Healthcare, Buckinghamshire, UK).

### Statistical analysis

Mean values were statistically compared using the Mann–Whitney U test. *P* values < 0.05 were considered statistically significant.

## RESULTS AND DISCUSSION

### PF-00477736, Chk1 siRNA and MK-1775 treatments abrogate radiation-induced G2 arrest in HeLa-Fucci cells

**Fig. 1.**
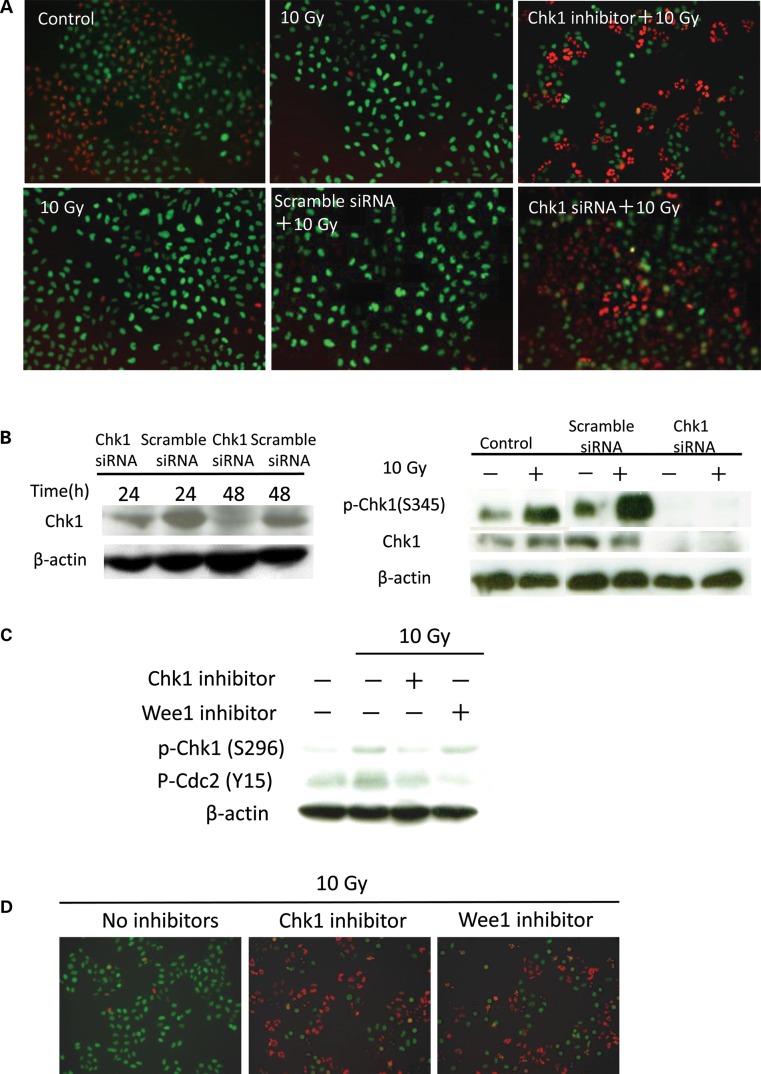
Effects of PF-00477736, Chk1 siRNA, and MK-1775 on radiation-induced G2 arrest in HeLa-Fucci cells. (**A**) PF-00477736 and Chk1 siRNA abrogated radiation-induced G2 arrest to similar extents in HeLa-Fucci cells. Cells were treated with 300 nM PF-00477736, 5 nM Chk1 siRNA, or 5 nM scramble siRNA, and then irradiated at a dose of 10 Gy. PF-00477736 was added 10 min before irradiation; siRNA-treated cells were transfected 48 h before irradiation. Representative images taken 14 h after irradiation are shown. The experiments of irradiation alone were performed simultaneously together with combined treatment of Chk1 inhibitor (upper) or siRNA (lower). (**B**) Western blotting to detect Chk1 and the phosphorylated form of Chk1 after siRNA transfection. Left panel: cells were transfected with 5 nM scramble or Chk1siRNA and lysed 24 or 48 h after transfection. Right panel: cells were transfected with 5 nM siRNA and irradiated 48 h after transfection; 2 h after irradiation, cells were lysed and prepared for Western blotting. Extra lanes between control and scramble siRNA were deleted from this panel. (**C**) Western blotting to detect the phosphorylated form of Chk1 (S296) and Cdc2 (Y15) after treatment with Chk1 or Wee1 inhibitor. Cells were lysed and prepared for Western blotting 18 h after irradiation with or without Chk1 or Wee1 inhibitor. (**D**) PF-00477736 and MK-1775 abrogated radiation-induced G2 arrest to similar extents in HeLa-Fucci cells. Cells were treated with 300 nM PF-00477736 or 60 nM MK-1775, and then irradiated at a dose of 10 Gy. Representative images were taken 18 h after irradiation.

Several kinds of Chk1 inhibitors have been tested and shown to abrogate radiation-induced G2 arrest [1]. PF-00477736 is a potent ATP-competitive inhibitor of Chk1; in previously reported experiments, the compound was used at a concentration ≥150 nM [6]. We have shown that HeLa-Fucci cells clearly enter G2 arrest after 10-Gy irradiation, reflected by the accumulation of green cells peaking ∼15 h after irradiation and their subsequent gradual disappearance [4, 5]. We found that 300 nM PF-00477736 clearly abrogated the accumulation of green cells, and observed similar effects after Chk1 siRNA treatment (Fig. 1A). Chk1 siRNA treatment significantly reduced levels of Chk1 and p-Chk1, whereas no such effect was observed in cells treated with the control siRNA (Fig. 1B). Thus, we concluded that the observed G2 arrest after PF-00477736 treatment was due to specific inhibition of Chk1 activity. Wee1 is also associated with DNA damage-induced G2 arrest [7, 8]. We examined inhibition of phosphorylation of Cdc2 at Tyr15 following combined treatment with inhibitor of Chk1 or Wee1 (MK-1775) and irradiation; the latter compound resulted in stronger inhibition (Fig. 1C). This was consistent with the results of previous reports (7, 8). Inhibition of Chk1 activity by the Chk1 inhibitor was confirmed by abrogation of Chk1 phosphorylation at Ser296 (Fig. 1C). In addition, we observed that Wee1 inhibitor almost completely abolished the radiation-induced G2 arrest: many red cells were observed 18 h after irradiation (Fig. 1D), as detected by the Fucci system for the first time.

### Pedigree analysis of HeLa-Fucci cells after irradiation with or without Chk1 inhibitor

**Fig. 2.**
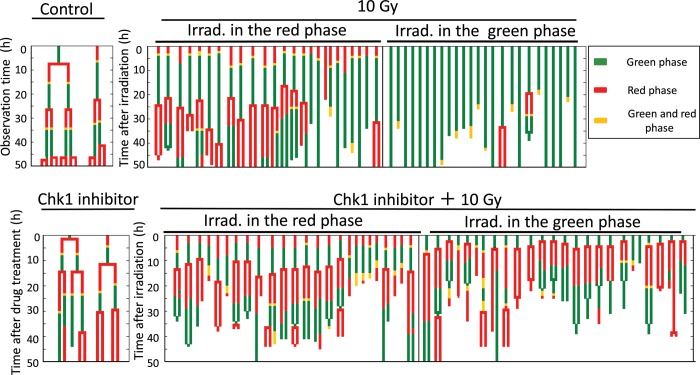
Pedigree assay of HeLa-Fucci cells using time-lapse imaging. Time course of fluorescence color change, cell division, and cell fusion is shown for 25 randomly selected cells in each group (i.e. cells irradiated with or without Chk1 inhibitor, as described in Fig. 1). Cells were analyzed separately depending on whether they were irradiated in the green or red phase. Lines ending within 50 h represent cell death at the indicated times. The two cells shown as controls were not exposed to irradiation or drug treatment. The two cells shown as ‘Chk1 inhibitor’ were exposed to drug treatment alone.

**Fig. 3.**
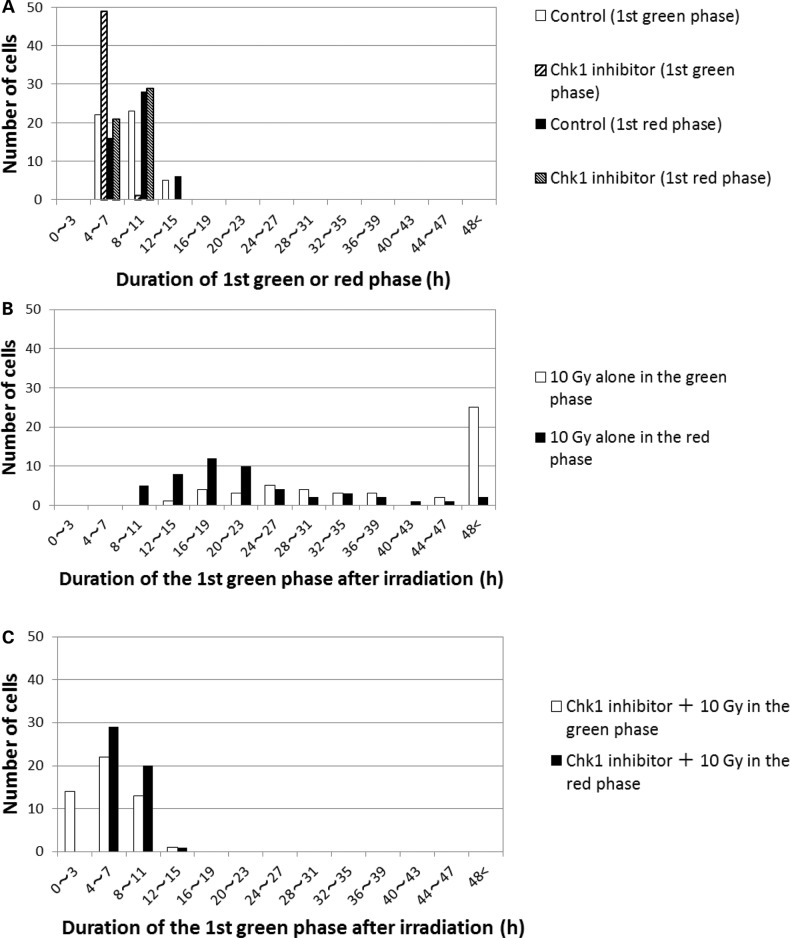
Effects of Chk1 inhibitor on the distribution of the first green phase duration (FGPD) after irradiation: (**A**) Chk1 inhibitor alone. (**B**) 10-Gy irradiation alone. (**C**) 10-Gy irradiation plus Chk1 inhibitor. In A, the first green- or red-phase durations were determined with or without Chk1 inhibitor treatment. In B and C, the distributions of cells irradiated in the red and green phases differ significantly (*P* < 0.01). The first red-phase durations (in A only) and FGPDs were measured from pedigree assays performed on 50 randomly selected cells.

The Fucci system permits differential analysis of cells irradiated in the red or green phase under live conditions, which cannot be achieved by flow-cytometric analysis. Figure 2 shows a pedigree analysis of cells irradiated either alone or in the presence of Chk1 inhibitor (25 cells in each group); the cells were differentially analyzed depending on the cell-cycle phase during which they were irradiated. Many cells irradiated in the red phase divided after irradiation, whereas very few cells irradiated in the green phase divided within the 50 h after irradiation. This finding indicates that cells irradiated in G1 phase enter M phase more rapidly than cells irradiated in other phases (Fig. 2). This was revealed for the first time by the Fucci system. Furthermore, we often observed mitotic catastrophe associated with abnormal expression of both fluorescent signals at M phase in cells irradiated in the green phase [9]. To precisely analyze the duration of G2 arrest, we obtained the distribution of the first green phase duration (FGPD) from pedigree analysis on independent sets of 50 cells each (Fig. 3). When cells were treated with Chk1 inhibitor alone, FGPD was somewhat shortened, but the first red-phase duration was not changed (Fig. 3A). Thereafter, however, both red- and green-phase durations tended to increase (Fig. 2). When cells received irradiation alone, FGPD differed depending on the phase at which irradiation took place: cells in the green phase at the time of irradiation exhibited a significantly longer duration (modal value: > 48 h) than those in the red phase (modal value: 20–23 h) (*P* < 0.01), even considering that in the former case, the duration was measured from the middle of the phase. Karanam *et al*. reported that DNA double-strand breaks (DSBs) generated in G1 phase are promptly repaired via nonhomologous end joining (NHEJ), whereas those in S phase are repaired more slowly because it takes time for the choice of repair mechanism between homologous recombination and NHEJ [10]. Thus, we speculate that cells irradiated in the red phase were released from G2 arrest more quickly than those irradiated in the green phase. Cell fusion was rarely observed. Treatment with Chk1 inhibitor prior to irradiation significantly reduced the FGPD in cells irradiated in either phase (almost to the control range observed in unirradiated cells ( < 15 h)). Furthermore, we often observed cell fusion in Chk1 inhibitor-treated irradiated cells (Fig. 2; fused cells are indicated by bifurcated traces that later join together).

## CONCLUSION

In this study, we found that 10-Gy irradiation induces a significantly elongated G2 arrest when cells are irradiated in the green phase, relative to cells irradiated in the red phase. Furthermore, we revealed for the first time that Chk1 inhibition abrogates the duration of G2 arrest to a similar extent regardless of the cell-cycle phase at the time of irradiation. Although the molecular mechanism remains to be elucidated, these results provide clear information regarding the time dimension of the effects of Chk1 inhibition on radiation-induced G2 arrest.

## FUNDING

This study was supported in part by Grants-in-Aid for Scientific Research from the Ministry of Education, Culture, Sports, Science and Technology (MEXT) and the Japan Society for the Promotion of Science (JSPS) (252855, 23390427 and 25670796) to A.K. and M.M. Funding to pay the Open Access publication charges for this article was provided by MEXT.
